# Forecasting Covid-19 in the United Kingdom: A dynamic SIRD model

**DOI:** 10.1371/journal.pone.0271577

**Published:** 2022-08-10

**Authors:** Gustavo M. Athayde, Airlane P. Alencar

**Affiliations:** 1 INSPER - Institute of Education and Research, São Paulo, SP, Brazil; 2 São Paulo School of Economics, EESP/FGV, São Paulo, SP, Brazil; 3 Institute of Mathematics and Statistics, University of São Paulo, São Paulo, SP, Brazil; Hodeidah University, YEMEN

## Abstract

Making use of a state space framework, we present a stochastic generalization of the SIRD model, where the mortality, infection, and underreporting rates change over time. A new format to the errors in the Susceptible-Infected-Recovered-Dead compartments is also presented, that permits reinfection. The estimated trajectories and (out-of-sample) forecasts of all these variables are presented with their confidence intervals. The model only uses as inputs the number of reported cases and deaths, and was applied for the UK from April, 2020 to Sep, 2021 (daily data). The estimated infection rate has shown a trajectory in waves very compatible with the emergence of new variants and adopted social measures. The estimated mortality rate has shown a significant descendant behaviour in 2021, which we attribute to the vaccination program, and the estimated underreporting rate has been considerably volatile, with a downward tendency, implying that, on average, more people are testing than in the beginning of the pandemic. The evolution of the proportions of the population divided into susceptible, infected, recovered and dead groups are also shown with their confidence intervals and forecast, along with an estimation of the amount of reinfection that, according to our model, has become quite significant in 2021. Finally, the estimated trajectory of the effective reproduction rate has proven to be very compatible with the real number of cases and deaths. Its forecasts with confident intervals are also presented.

## Introduction

The first cases of covid-19 occurred in Wuhan, China, and they were reported as a viral pneumonia. In January, 2020, the Chinese government confirmed an outbreak caused by a novel coronavirus. The pandemic was disseminated all around the world.

We analysed the daily number of new registered cases of covid-19 and deaths from April, 2020 to September, 2021 in the United Kingdom. On 31th January, 2020, there were only two confirmed cases of covid-19 [1] and the first death occurred on Mar 6th, 2020, when there were 374 registered cases. By the end of March, the region had already accumulated 38,815 cases and 2,457 deaths, with 4,534 cases and 404 deaths registered only on March 31th, 2020. Through the whole sample, the daily number of cases and deaths presented at least two waves, where the largest daily number of deaths occurred on January 27th, 2021, with 1,726 deaths.

During the pandemic, it is reasonable to assume that the mortality and infection rates oscillate over time due to several distancing measures [2] (which were imposed and relaxed), new variants [3] and due to the introduction of vaccination. Historically, the epidemics may be analysed dividing the population into compartments (susceptible-infected-recovered) as in the well-known SIR Model [4] and its extension including deaths, called SIRD model. In essence, at every moment the population is divided into susceptible, infected, recovered, and dead. As time goes by, part of the susceptible individuals may be infected and these infected individuals may become recovered or dead. This model was used for the covid infection in [5]. Some extensions include other compartments, as vaccinated and quarantined, and even spatial and age-group structure. These and a complete review of other models to surveillance of infectious diseases are discussed for example in [6].

The usual SIRD model assumes that the infection, mortality and recovered rates are constant over time. This is a too strong assumption that may not be valid because the dynamics of the spread of this covid-19 epidemic depends on several factors that are changing over time. For example, incorporating the habit of wearing masks, lockdowns and social distance restrictions may affect the infection rate; advances in treatments may impact the mortality and recovery rates and a health system collapse may increase considerably the mortality rates. Even the perception of the population about the severity of the epidemics may affect the prevention measures, and ultimately these rates.

The main contribution of our proposal is to allow some of these rates, which drive the epidemic dynamics, to change over time depending on the corresponding rate in the previous moment, as a Markov process. This dynamic SIRD model is written as a non-linear state space model, which allows us to predict and forecast all the time-varying rates with their corresponding confidence intervals. Also, the effective reproduction number (*R*_*e*_(*t*)), that measures the mean number of individuals who will be contaminated by each infected individual, is also changing over time and will be predicted with its corresponding interval.

Another novelty of our proposal is to include the undereporting rate, which also changes with time. After fitting this model, it is possible to estimate the well-known effectively reproduction number, that measures the average number of people that will be contaminated for each infected person.

All results are shown for the United Kingdom, which presented different strategies to control the epidemics and a time-varying dynamics.

The use of state-space models in the SIRD models had already been done before. However, in a much more restricted manner than the one we are proposing in this paper. They have chosen to model solely the number of susceptible, infected, recovered and dead people, as non-observed (state) variables, so there would be a noise component in measuring each of these variables. We have also done that. However, since they assumed that all the rates are fixed (as opposed to our case, where they vary over time), the former models focused on simply capturing (and cleaning) the noises of S, I, R. This first representation did not provide substantial gains in terms of adding flexibility to the model. Examples of these approaches can be found in [7, 8] and more recently, relating to Covid-19, we have [9, 10]. Other new approaches generalize the SIRD model by assuming that each observation is composed of different waves, each one following the traditional SIRD dynamics. In order to properly estimate the parameters, the assumption used was that the number of infected individuals would follow a gaussian or gamma mixture model [11, 12]. These approaches allow the discrimination of the effect of each wave in the SIRD compartments. One aspect that was not pointed out in all these former works was a possible interpretation when the random effect made the Susceptibles compartment increase and the Recovered compartment decrease (which is impossible in the original SIR system). In this paper, we have captured this behaviour as well, and we interpreted this phenomenon as reinfection (which the original SIR does not allow either).

As already mentioned, not only we have followed the former works with a new interpretation of reinfection, but also we have allowed the infection and mortality rates to change over time. The recovery rate is assumed to be fixed, for there seems to be no significant advances in the treatments to diminish the disease cycle so far.

The next section describes the main issues of the proposed model and some pragmatic suggestions to be applied when fitting the model for real data. After fitting the proposed model for daily data from the United Kingdom, all the predicted rates and forecasts are presented in the Results’ section. Finally, in the Discussion section, it is presented some associations between the estimated rates and some empirical facts, as lockdowns and variants of the coronavirus. Finally, some advantages and caveats of the proposed model are also discussed.

## Materials and methods

Daily time series of confirmed cases and deaths due to covid in the United Kingdom from April, 2020 to September, 2021 were obtained at the ourworldindata website, specifically at https://ourworldindata.org/coronavirus/country/united-kingdom. The moving average of seven days were calculated for all daily time series to eliminate or reduce the weekly seasonality, caused by less registered cases and deaths in weekends and holidays. All models were fitted to these smoothed series of 7-days moving averages of new cases and deaths. All forecasts are presented from October to December, 2021.

The estimated population is estimated as 67,886,004 for the United Kingdom in 2020 and the population is assumed to be constant over time (with no migration).

In the original epidemic susceptible-infectious-recovered (SIR) model, the population is divided into three compartments. We may consider that some of the infectious people will recover, but some will decease, then the third compartment may be called “removed”, and this compartment includes both recovered cases and deaths. By adding the mortality rate, the SIRD model is able to divide the fraction who died and that recovered. We present our proposed dynamic SIRD model as

$$\begin{array}{ccc} S_{t} & = & S_{t-1}-\beta_{t}S_{t-1}I_{t-1}, \end{array}$$ (1)

$$\begin{array}{ccc} I_{t} & = & I_{t-1}+\beta_{t}S_{t-1}I_{t-1}-\gamma I_{t-1}, \end{array}$$ (2)

$$\begin{array}{ccc} R_{t} & = & R_{t-1}+\gamma I_{t-1}\left(1-\mu_{t-1}\right), \end{array}$$ (3)

$$\begin{array}{ccc} D_{t} & = & D_{t-1}+\gamma I_{t-1}\mu_{t-1}, \end{array}$$ (4)

where *S*_*t*_, *I*_*t*_, *R*_*t*_, and *D*_*t*_ stand respectively for the proportion of susceptible, infected, recovered individuals, and deaths; *β*_*t*_ is the time-varying disease infection (transmission) rate; *μ*_*t*_ is the time-varying mortality rate; and *γ* is the removed rate, which is assumed to be constant over time, and the “removed” compartment consists of recovery cases and deaths. The effective reproduction number is *R*_*e*_(*t*) = *β*_*t*_*S*_*t*_/*γ* [13].

In contrast to some previous papers, in our model we have made use of a logit transformation to ensure that each rate is bounded in the interval [0, 1]. The mortality rate (*μ*_*t*_) was restricted to the interval [0, 0.05], since it is not feasible to observe higher rates. Therefore, it is assumed the following reparametrization

$$\begin{array}{ccc} S_{t} & = & \frac{e^{s_{t}}}{1+e^{s_{t}}+e^{i_{t}}}\;\Rightarrow s_{t}=\text{ln}\left(S_{t}\right)-\text{ln}\left(1-S_{t}-I_{t}\right)\\ I_{t} & = & \frac{e^{i_{t}}}{1+e^{s_{t}}+e^{i_{t}}}\;\Rightarrow i_{t}=\text{ln}\left(I_{t}\right)-\text{ln}\left(1-S_{t}-I_{t}\right)\\ \alpha_{t} & = & \frac{e^{a_{t}}}{1+e^{a_{t}}}\\ \beta_{t} & = & \frac{e^{b_{t}}}{1+e^{b_{t}}}\\ \mu_{t} & = & 0.05\frac{e^{m_{t}}}{1+e^{m_{t}}}, \end{array}$$

where *α*_*t*_ is the reporting rate, *β*_*t*_ is the infection rate, and *μ*_*t*_ is the mortality rate.

The model begins with following two equations:

$$\begin{array}{ccc} \text{ln}(\frac{NC_{t}}{Pop}) & = & \text{ln}\left(\alpha_{t}\right)+\text{ln}\left(\beta_{t}\right)+\text{ln}\left(S_{t}\right)+\text{ln}\left(I_{t}\right)+w_{t}^{1} \end{array}$$ (5)

$$\begin{array}{ccc} \text{ln}(\frac{ND_{t}}{Pop}) & = & \text{ln}\left(\gamma \right)+\text{ln}\left(\mu_{t}\right)+\text{ln}\left(I_{t}\right)+w_{t}^{2}. \end{array}$$ (6)

where *Pop* is the population, which is assumed to be constant over time, *NC*_*t*_ is the number of new registered cases at time t, *ND*_*t*_ is the number of deaths at time t, and *w*_*t*_ is a zero-mean, time-independent, Gaussian random vector with contemporaneous covariance matrix W (2 × 2 matrix).

Now, we may state the complete specification of the state space model. The first two equations are called observation equations and now they are written taking into account the reparametrization. The state equations describe the dynamics of the non-observed variables.

$$\begin{array}{ccc} s_{t-1} & = & s_{t-2}+\text{ln}(1-\frac{\beta_{t}e^{i_{t-2}}}{1+e^{s_{t-2}}+e^{i_{t-2}}})-\text{ln}\left(1-\gamma e^{i_{t-2}}\right)+v_{1,t} \end{array}$$ (7)

$$\begin{array}{ccc} i_{t-1} & = & i_{t-2}+\text{ln}(1-\frac{\beta_{t}e^{s_{t-2}}}{1+e^{s_{t-2}}+e^{i_{t-2}}})-\text{ln}\left(1-\gamma e^{i_{t-2}}\right)+v_{2,t} \end{array}$$ (8)

$$\begin{array}{ccc} a_{t} & = & a_{t-1}+v_{3,t} \end{array}$$ (9)

$$\begin{array}{ccc} b_{t} & = & b_{t-1}+v_{4,t} \end{array}$$ (10)

$$\begin{array}{ccc} m_{t} & = & m_{t-1}+v_{5,t}, \end{array}$$ (11)

where *v*_*t*_ is a zero-mean, time-independent, Gaussian random vector with contemporaneous covariance matrix *V* (5×5 matrix). The vectors *v*_*t*_ and *w*_*t*_ are completely independent, even when one vector is lagged with respect to the other.

This final model is written as a non-linear state space model, with two observed variables: the daily number of new cases and the daily number of deaths. In the linear state space models, the non-observed variables (called state variables) are estimated using the Kalman filter [14, 15]. In our non-linear state space model, the predictions of all non-observed components, as each rate and the susceptible, infected, and recovered proportions were estimated using the Extended Kalman filter, which basically deals with a linearized approximations via Taylor expansion series of the original non-linear system [16, 17]. The Extended Kalman filter also provides the mean squared error of all predictions and forecasts and it is possible to calculate the 95% confidence intervals. All the model parameters are estimated by the maximum likelihood method.

## Results

**Fig 1:**
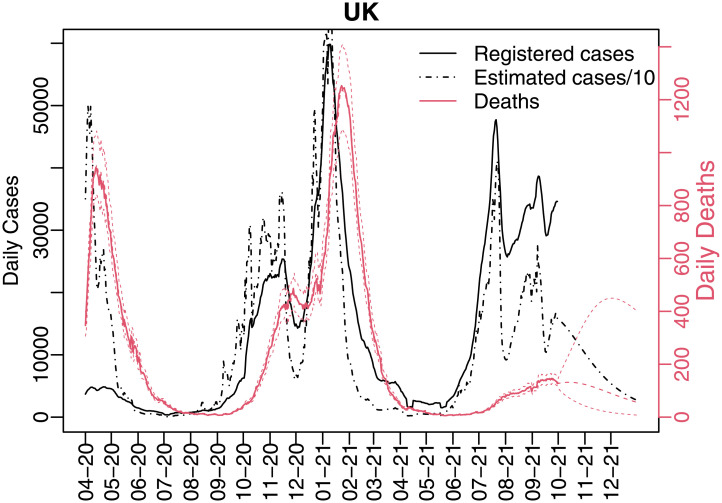
Daily 7-days moving average of new registered cases, estimated cases divided by 10 and deaths.

In the United Kingdom, the first case was registered in January, 2020 and the first death in March, 2021. In April, 2020, there were registered more than 1,000 daily deaths in nine days. Due to several restrictions, as lockdowns, the number of daily deaths decreased rapidly as can be seen in Fig 1. But the number of cases and deaths increased again by the end of 2020. On January 8th, 2021, the daily number of new cases of covid-19 reached 68,192 cases in the United Kingdom, with the 7-days moving average of almost 60,000 cases. Nineteen days later, the daily number of deaths reached its peak with 1,726 deaths. After February, 2021, the number of cases and deaths started to decrease sharply and, by July, 2021, only the number of cases started to grow again, while the number of deaths increased not at the same rate, maybe due to the vaccination program. The number of cases and deaths were observed until September, 2020, and the forecasts were calculated from October to December, 2021.

Considering the estimated underreporting rate *α*_*t*_, the estimated real number of cases is also presented in Fig 1 (we divided it by 10 to put it in a similar scale to the reported cases). The real number of cases may have reached 500,000 daily cases in the beginning of the epidemics, instead of only 5,000 registered cases. Also, we may have had 60,000 daily cases in the beginning of 2021 instead of 6,000 reported.

**Fig 2:**
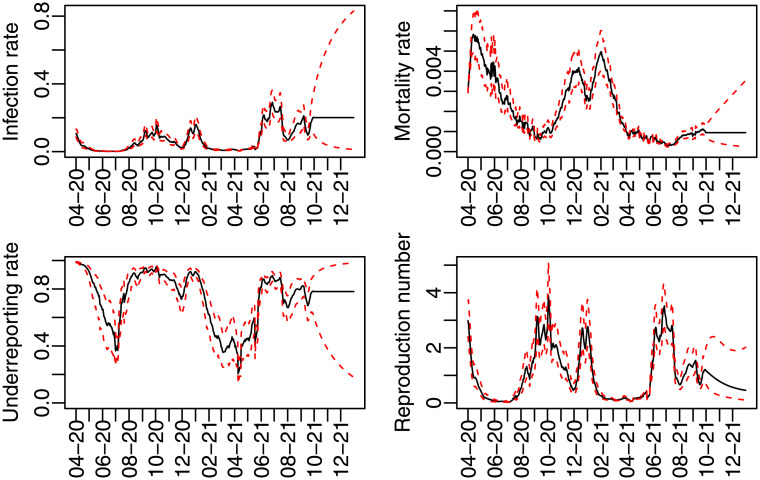
Time varying predicted infection, mortality, subnotification rates and effective reproduction number.

In Fig 2, the predicted infection rate is higher at the very beginning, in April 2020 and October 2020. It was lower in November 2020, but it increased again by the end of 2021 till it reached its peak in June and July of 2021. This captures well the behavior of the number of cases.

The mortality rate reached its highest value soon in April 2020, when the treatments and the health system were not prepared. It decreased until September, but it presented higher values during the winter, from Dec.2020 to Feb, 2021. It did not increase again even with the rise of the infection by June, 2021, probably due to the vaccination program. The mortality rate forecast for the end of 2021 is only 0.10% (95% C.I. = [0.03%; 0.37%]).

The underreporting rate oscillates around 85%, with lower values around June, 2020 and April, 2021. In September, 2021, the mean of this predicted rate is around 79%, with forecasts of 78% in the end of 2021. It is worth noticing that the underreporting rate is larger in the beginning of the considered period and is smaller in the second semester of 2021. This may also be seen in Fig 2.

Finally, Fig 2 presents the predicted effective reproduction number *R*_*e*_(*t*). If it is larger than one, an infected person is expected to transmit the covid-19 to more than one individual. The predicted *R*_*e*_(*t*) was greater than one in the following periods: in April 2020; from August to October 2020; from December 2020 to January 2021; June and July 2021; and by the end of September 2021. It is worth noticing that this predicted *R*_*e*_(*t*) was higher than 2 in October 2020 and December 2020 and it was only 0.5 by the end of November 2020, so it was oscillating considerably by the end of 2020. The forecast reproduction number is decreasing and it is expected to be less than one in October 2021 and 0.44 by the end of 2021.

**Fig 3:**
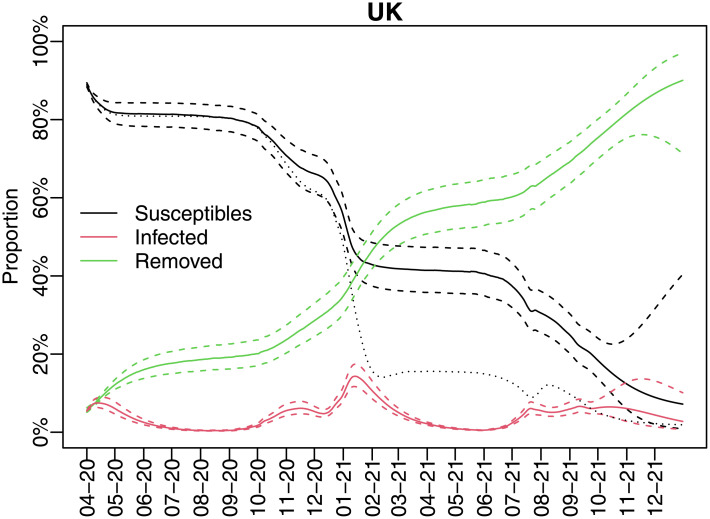
Predicted susceptible, infected and removed proportions (95% CI) and the susceptibles without reinfection (dotted line).

The population proportion in each compartment, namely susceptible, infected, and removed, is predicted over time and all proportions are depicted in Fig 3. The infected proportions present several peaks reaching its maximum at 15% in the beginning of 2021. The forecast proportion of infected population goes from 6% in October 2021 to 2% (95% C.I. = [1%, 7%]) by the end of 2021.

The proportion of susceptibles dropped from 80% in October 2020 to 40% in January 2021. After a period of stabilization, the susceptible population decreased further after July, it became 16% at the end of September, and its forecast value is only 6% at the end of the year. As expected, removed population increased and it is expected to be 92% by the end of 2021.

Considering the same model but without the reinfection, the susceptibles’ proportion would be less than 20% in the beginning of 2021, which could be unrealistic. Another evidence in favor of the reinfection hypothesis is that the random shocks of the state variables *s*_*t*_ and *i*_*t*_ were essentially positive and highly correlated. Therefore, the shocks were essentially transferring from the recovery compartments to the susceptible and infected compartments.

The removed rate (*γ*) was estimated as 1/31, indicating that an average time of 31 days is necessary for the covid-19 recovery or time until death.

## Discussion

The predicted rates have varied considerably over time, thus capturing the different stages of the covid-19 epidemic in the United Kingdom. The first stage consists of a larger predicted mortality rate and large effective reproduction number on April 2020, with a not yet very large number of registered cases.

The first lockdown was announced on March 23th 2020 in the United Kingdom [2]. On May 10th, the prime minister said that only people who could not work from home should return to workplace, but avoiding public transport. In June, schools and non-essential shops started to reopen. Probably this lockdown was responsible to reduce the infection rates and, consequently the number of cases, as seen in Figs 1 and 2.

Only on Sep. 22nd, new restrictions were imposed, but a second national lockdown only occurred in November 2020, and a third one in January 2021. Only in February 2021 the infection rate was low and the mortality rate definitely started to decrease. In April 2021, all the non-essential services were reopened.

The number of daily cases and the predicted infection rate was again higher in July 2021. Nevertheless, the mortality rate maintained a lower level. This may be a consequence of the vaccination program, which started in 2021 [18]. On May 1st, 60% of population aged 12 and over have already received the first dose of the covid-19 vaccine and 27% received the second dose. On July 1st, 78% of population over 12 years old received the first dose and 57% already received two doses. On September 1st, these percentages increased respectively to 84% and 75%. The vaccination may explain this control of mortality, despite the growing number of cases. Also, it is worth noticing that a third dose vaccination began in October 2021.

Another relevant issue of the covid-19 pandemic was the emergence of new variants of the coronavirus. A very complete study of all variants of the coronavirus until 2021 and its main features related to the molecular structure, transmission, and dissemination around the world is presented in [3]. These variants are considered variants of concern when they show evidence of an increase in transmissibility, disease severity, and a significant reduction in neutralization by antibodies generated during previous infection or vaccination. Following [3], we may point out some important variants that have played a significant role in the UK up to September 2021. The Alpha variant was identified in the UK in November 2020 and its associated mortality was 55% higher than the original, and was predominant in the second wave. Later, the Delta variant (which was first identified in Maharashtra, India, in October 2020), was declared as responsible for the third wave in the UK, where it seemed to be around 60% more transmissible than the Alpha variant. This behaviour appears to be quite consistent with our estimated trajectory of the infection rate (*β*_*t*_) in Fig 2.

The vaccine efficacy may be reduced for some variants. Pfizer-BioNTech and Moderna mRNA COVID-19 vaccines seem to be effective in symptomatic alpha variant infections [3], but the majority of the vaccines have lower efficacy against beta (which was identified in South Africa) and delta variants [3]. [19] concluded that vaccination with the Pfizer-BioNTech and Oxford- Astrazeneca still reduces new infections, but their effectiveness are reduced with the delta variant. Another relevant point is that the people immunized against COVID-19 would lose approximately half of their defensive antibodies every 108 days or so [20]. Regarding reinfection, [21] forecast a 50% risk of reinfection 17-months after a first infection, without the recommended restrictions and vaccination. According to [22], COVID-19 vaccines seem to be effective at preventing most infections in the United States. In the case of the delta variant, it played a significant role in avoiding severe disease and hospitalization. The estimated trajectory of the mortality rate in Fig 2 seems to suggest that this is probably also occurring in the UK.

Despite the large proportion of vaccinated people, the rise of new covid cases since July 2021 may have occurred due to this reduced vaccine effectiveness with regard to infections and also a higher transmission. According to [23], the vaccines alone are not able to avoid infections, and this observed rise would have been caused also by relaxing all restrictions, such as social distancing and the use of masks.

All these changing conditions justify models with time-varying parameters. A few models were developed to deal with this issue, fitting deterministic functions. [24] proposed a model where the infection, recovery, and mortality rates change over time, but the time-varying parameters are written as linear combinations of functions that form a basis for the function space. They present the estimated rates in Italy from February to May 2020 and they found monotone decreasing infection and mortality rates, and an increasing linear recovery rate. In [25] it was assumed a functional form of a time-dependent infection rate, where this rate was constant before the lockdown, and it gradually decreases after the lockdown and other adopted containment measures. An interesting aspect of this methodology is the ability to evaluate the effect of the lockdown and other restrictions. In order to include the effect of social distancing measures, [26] proposed to replace the number of susceptible individuals by a non-linear function of *S*_*t*_ that depended on the social distance adopted at time t. Other strategies were made based on dividing the considered sample period into stages and change the parameters depending on each stage, as in [27, 28].

Other approaches that dealt with time-varying rates incorporating stochastic frameworks were made also. In [29], after generating a historical series of the Reproduction rate (based on the number of reported cases and by assuming a constant recovery rate of 1/7), a Kalman Filter was used to smooth this historical series. [9] consider the quarantine effects in the SIR model using a Bayesian approach. Unlike our work, the number of deaths was not taken into account in these two latter studies.

One of the main advantages of our proposed model is the flexibility in which the time-varying rates evolve, and the possibility to work with this wide range of different variables that change over time, like the the possibility of reinfection, the inclusion of the reporting rate, and the estimation of all rates and forecasts (including the effective reproduction number) with their corresponding confidence intervals.

The time-varying rates were able to capture the dynamic evolution of all restrictions, the resumption of all activities, vaccination, variants, and other non-controlled factors. Also, our proposed model allows for reinfection (in a subtle way), since a recovered person may become susceptible and infected again.

The inclusion of the underreporting rate, changing over time, may capture that testing has been primarily restricted to individuals with moderate to severe symptoms due to limited test availability [30]. [30] estimates 6,454,951 cumulative infections compared to 721,245 confirmed cases in the United States on April 18th, 2020. This corresponds to an underreporting rate of 89%, which is similar to the predicted rates we found for the UK, as seen in Fig 2. Based on ordered and complete covid tests and on test sensitivity, another study estimated that 1 in 7.7 (13%) of total infections were identified and reported [31].

The main caveat of our model, in terms of forecasting, is that it provides wide out-of-sample confidence intervals, which although is an expected behaviour (due to the volatility of the estimated random variables) it does not bring confident predictions for the long run. The forecast effective reproduction number is 0.44 by the end of the year 2021 and the forecast proportion of infected population is 2% (95% C.I. = [1%, 7%]) by the end of 2021. In general, the traditional SIRD model describes a pandemic that evolves as a single wave. The infected compartment grows up to a peak, and diminishes until it vanishes. In the end of the pandemic, the susceptibles end up flattening in a lower value than the original level, and the removed ones finally stabilize at a higher value than in the beginning. This behaviour was not observed in the Covid pandemic, which had shown several waves with different shapes. Our model was able to capture this complex behaviour due to the flexibility we brought to the dynamics of its parameters and variables. The evolution of the compartments, dealing with reinfection, and multiple waves seem to fit quite well the evolution of the pandemic in UK, as can be seen in Fig 3.

Another concern when fitting the model is to avoid local likelihood maximum points, which requires a more intensive computing processing, initializing the optimization routine with different initial points.

For some future extensions of our framework, the most natural seems to be incorporating more compartments in the family of the SIR models, like hospitalizations and Intensive Care Unit occupation. Each new compartment would require a new observation equation. Apparently there would be no need to add underreporting rates to these observations. The underreporting rate seems to be significant only in terms of the number of cases reported.

Another useful development of our work would be to incorporate the reinfection rate as a proper state variable in a more explicit manner (we have basically inferred reinfection by the behavior implied in the random shocks of the compartments). Nevertheless, adding a (dynamic) term that would throw recovered individuals back to the susceptible compartment could bring some complications in the estimation process in terms of identification of variables.

Finally, we have chosen to estimate the system via maximum likelihood assuming normal distributions. Other distributions (especially fat-tailed ones) would also be welcomed to verify the robustness of our results. In the same manner, it would be interesting to compare other approaches to deal with the non-linearity in the Kalman Filter rather than the Extended Kalman Filter.

## Conclusion

We have proposed a state-space framework for the SIRD model in which the infection, mortality and underreporting rates become time-varying stochastic parameters. Reinfection estimates have become possible as well, due to the way we modelled the evolution of the Susceptible-Infected-Recovered-Dead compartments. Using only reported cases and deaths numbers, all the trajectories and forecasts were estimated with confidence intervals. The model was fitted to the UK covid data from April 2020 to September 2021.

The estimated trajectory of the infection rate matched very well the waves of new variants and social restrictions. The significant decrease along 2021 of the mortality rate suggests the vaccination program was quite effective in reducing deaths. The estimated underreporting rate, although quite high and very volatile, has shown a downward trend, and a tendency to decrease in the most stressed periods, as expected. We have found evidence that reinfection became quite significant in 2021. The evolution of the effective reproduction rate was also very consistent with the historic data.

Finally, although we provide forecast for all the variables for the future months, it is quite remarkable how the confidence intervals become rapidly wide the further we go, leaving not much room for confident predictions.

## Abbreviations

*D*_*t*_: Proportion of accumulated Deaths until time t
*I*_*t*_: Proportion of Infected individuals at time t
*NC*_*t*_: Number of new cases at time t
*ND*_*t*_: Number of new deaths at time t
Pop: Population
*R*_*e*_(*t*): Effective Reproduction number
*R*_*t*_: Proportion of Recovered individuals at time t
*S*_*t*_: Proportion of Susceptible individuals at time t
SIRD model: Susceptible-Infected-Recovered-Death model
*α*_*t*_: Reporting rate
*β*_*t*_: Infection rate
*γ*: Removed rate
*μ*_*t*_: Mortality rate
